# A role for aquaporin (Aqp1) in the control of *Cryptococcus neoformans* cell morphology

**DOI:** 10.1016/j.jbc.2026.113444

**Published:** 2026-08-13

**Authors:** Piotr R. Stempinski, J. Alberto Patiño-Medina, Francisco G. Hernandez, Isabel A. Jimenez, Samuel Rodrigues dos Santos Junior, Peter Agre, Arturo Casadevall

**Affiliations:** 1 Department of Molecular Microbiology and Immunology, The Johns Hopkins Bloomberg School of Public Health, Baltimore, Maryland, USA; 2 Department of Molecular and Comparative Pathobiology, The Johns Hopkins University School of Medicine, Baltimore, Maryland, USA

**Keywords:** Cryptococcus, aquaporin, Cryptococcus neoformans, Titan cells, Fungal cell biology, Cryptococcal vacuoles, Polysaccharide capsule, ROS

## Abstract

Aquaporins are small, integral membrane channels that facilitate the transport of water across cellular membranes and, in the case of aquaglyceroporins, can also conduct specific neutral solutes, such as glycerol. These proteins are conserved across biological kingdoms, yet their roles in fungal physiology remain relatively understudied. In *Cryptococcus neoformans*, an opportunistic fungal pathogen, we examined the organism’s single aquaporin, Aqp1, and uncovered unanticipated influences on cellular morphology. Loss of Aqp1 resulted in smaller cells, whereas its presence promoted the formation of enlarged titan-like cells. This shift in size was closely linked to intracellular redox physiology. The overexpression of the cryptococcal aquaporin increased sensitivity to oxidative stress and led to the largest titan-like cells; antioxidant supplementation suppressed this enlargement, consistent with a ROS-dependent regulatory mechanism. Additionally, Aqp1 overexpression produced vacuolar abnormalities in titan-like cells, suggesting that excessive water influx strained intracellular organization during rapid cell expansion. These findings position Aqp1 at a functional crossroads connecting membrane transport, oxidative balance, and size control, and they support a model in which an aquaporin contributes to morphological plasticity by affecting intracellular redox physiology, which in turn allows *C. neoformans* to adapt to environmental pressures.

Aquaporins are a conserved family of transmembrane protein channels that govern the transport of water and, in some cases, small molecules such as glycerol, urea, and ammonia across biological membranes (1, 2, 3, 4). These channels play important roles in maintaining osmotic balance, regulating intracellular turgor pressure, and mitigating responses to external stress factors (1, 2, 5, 6). Aquaporins can be categorized into two classes: orthodox aquaporins, which are highly selective for water, and aquaglyceroporins, which allow the transport of water and some small uncharged molecules (1, 5, 7, 8). This functional diversity reflects their wide-ranging physiological roles across all kingdoms of life (3, 6, 7, 9, 10, 11). In fungi, aquaporins have been implicated in development, morphogenesis, freeze tolerance, sporulation, and responses to osmotic and oxidative stress (12, 13, 14). However, the roles of aquaporins in regulating virulence-associated processes of pathogenic fungi remain largely unexplored.

*Cryptococcus neoformans* is an opportunistic fungal pathogen and the etiologic agent of cryptococcosis, which is a life-threatening pulmonary and central nervous system infection, particularly in immunocompromised individuals (15, 16, 17, 18, 19). Cryptococcosis accounts for an estimated 180,000 deaths annually, disproportionately affecting populations with high prevalence of advanced HIV infection. The success of *C. neoformans* as a pathogen is primarily attributed to its ability to withstand and adapt to hostile host environments through diverse stress response mechanisms, production of multiple virulence factors, immune evasion strategies, and morphological plasticity (16, 20, 21, 22, 23, 24, 25, 26).

A key morphological feature of *Cryptococcus* spp. is a polysaccharide capsule, a major virulence factor that surrounds both typical and morphologically specialized yeast cell forms (19, 27, 28, 29). The capsule plays a critical role in protecting the fungus from host immune defenses, facilitating immune evasion and persistence during infection (19, 30). Among the various morphological adaptations observed in host cells during infection, the formation of titan cells represents a striking example of cryptococcal morphological plasticity (17, 31). These enlarged, polyploid cells are characterized by thickened cell walls, expanded capsules, and altered surface properties that enhance resistance to phagocytosis and increase tolerance to oxidative and nitrosative stresses (27, 32, 33). Titan cells are thought to modulate host immune responses and contribute to fungal survival and dissemination (27, 34, 35). Despite their importance in the pathogenesis of cryptococcosis, the molecular mechanisms driving titan cell formation remain incompletely understood.

In this study, we characterize the function of Aqp1, the single aquaporin identified in *C*. *neoformans*, through a combination of phenotypic, morphological, and structural analyses (12). Our studies reveal that Aqp1 can influence cell size and stimulate the formation of titan-like cells in *C. neoformans* in a ROS-dependent manner. Furthermore, we confirmed that Aqp1 participates in maintaining intracellular redox balance and distribution of reactive oxygen species within the cell. This connection between water channel function, oxidative homeostasis, and morphogenesis suggests that aquaporins contribute to the adaptive flexibility that allows *C. neoformans* cells to remodel their cellular morphology and size to form titan cells in response to environmental stressors.

## Results

### Structural prediction and homology analysis of cryptococcal Aqp1

**Figure 1:**
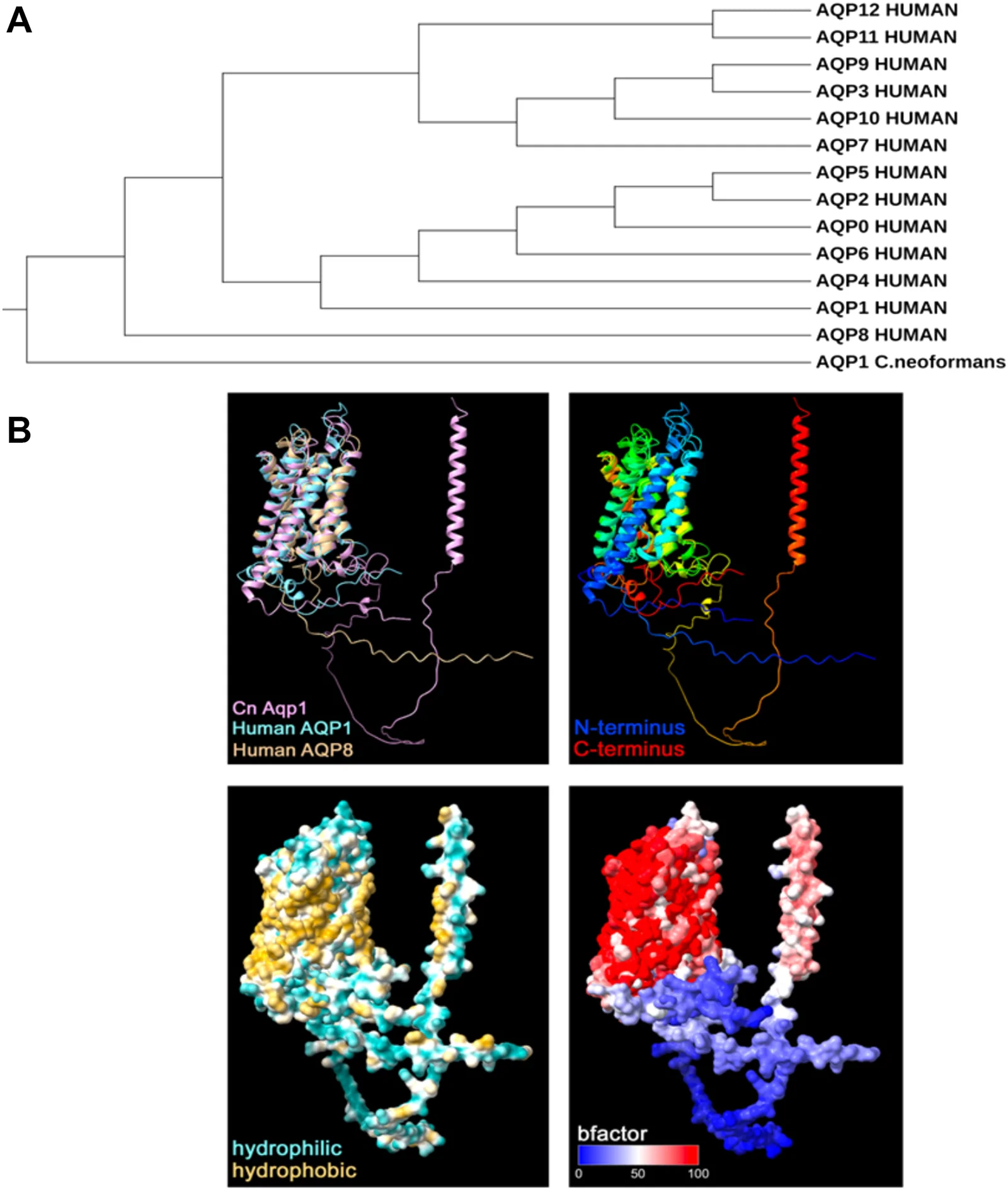
**Evolutionary comparison and structural modeling of *Cryptococcus neoformans* Aqp1.***A*, Phylogenetic analysis comparing the amino acid sequence of *C. neoformans* Aqp1 with human aquaporins, highlighting evolutionary relationships and potential functional similarities. *B*, AlphaFold predictive modeling of Aqp1 revealed a classical aquaporin fold, comprising six transmembrane helices and two conserved NPA (Asn-Pro-Ala) motifs, consistent with canonical water channel proteins. Structural alignment using ChimeraX demonstrated high structural homology between cryptococcal Aqp1 and its closest human counterparts, AQP8 and AQP1, including conserved positional alignment and similar surface hydrophobicity and electrostatic profiles.

To explore evolutionary conservation and potential functional similarities, we compared the amino acid sequence of *C. neoformans* Aqp1 with human aquaporins. BLASTp analysis identified the highest similarity between cryptococcal Aqp1 and human AQP8, a mitochondrial aquaporin involved in water and small solute transport (Fig. 1*A*) (36, 37, 38). AlphaFold predictive modeling of the secondary structure of cryptococcal Aqp1 revealed a classical aquaporin structure composed of six transmembrane helices and two conserved NPA (Asn-Pro-Ala) motifs, consistent with canonical water channel proteins (Fig. 1*B*) (1, 39). ChimeraX structural alignment revealed high structural homology, both in positional alignment and in surface hydrophobicity and electrostatic charges, between the cryptococcal Aqp1 and its human homologs (Fig. 1*B*). However, cryptococcal Aqp1 has an additional C-terminal domain that is not present in its human homologs. The existence of the C-terminal transmembrane domain is not conserved, and its presence is variable in different fungal species (Fig. S1). Thus, the predicted structure supports the functional classification of Aqp1 as a member of the aquaporin family, but also reveals an additional domain, absent from human aquaporin homologs, that may have functional significance specific to *Cryptococcus* spp. Using predictive modeling, this C-terminal domain was predicted to be intracellular, rather than extracellular or transmembrane, and was not predicted to be a signal peptide. To further evaluate the presence of Aquaporin-like domain (PF00230) across clinically relevant fungi, we performed Ortholog group analyses of the proteomes from multiple species. Our analysis revealed considerable variation in the number of Aqp1 homologues among different fungi (Table 1). The presence of a single protein containing an aquaporin domain (PF00230), such as found in *C. neoformans*, is relatively uncommon but not unique, as other species such as *Cryptococcus gattii*, *Malassezia sympodialis*, *Candida parapsilosis*, or *Rhizopus microsporus*, also possess only one putative aquaporin. In contrast, several fungal species have more aquaporin homologues, including *Exophiala spinifera* with 13 homologues and *Cladophialophora bantiana* with 10 channels. These findings suggest that aquaporin gene copy number is highly variable across fungal taxa, potentially reflecting differences in ecological adaptation and physiology of diverse fungal species.

**Table 1:** Overview of selected clinically relevant fungal species and Aqp1 homologues

Division	Species	Strain	No. of homologues
Basidiomycota	*Cryptococcus gattii*	VGII R265	1
	*Malassezia sympodialis*	ATCC 42132	1
	*Trichosporon asahii*	CBS 2479	4
Ascomycota	*Aspergillus fumigatus*	Af293	3
	*Aspergillus nidulans*	FGSC A4	5
	*Aspergillus niger*	ATCC 1015	7
	*Blastomyces dermatitidis*	ATCC 26199	2
	*Candida auris*	B11220	1
	*Candida glabrata*	BG2	4
	*Candida parapsilosis*	CDC317	1
	*Cladophialophora bantiana*	CBS 173.52	10
	*Coccidioides immitis*	H538.4	2
	*Emergomyces africanus*	CBS 136260	2
	*Exophiala spinifera*	CBS 89968	13
	*Fonsecaea pedrosoi*	CBS 271.37	9
	*Fusarium solani*	FSSC 5 MPI-SDFR-AT-0091	5
	*Histoplasma capsulatum*	G184AR	2
	*Lomentospora prolificans*	JHH-5317	4
	*Microsporum canis*	CBS 113480	2
	*Paracoccidioides brasiliensis*	Pb03	2
	*Sporothrix schenckii*	1099-18	2
	*Stachybotrys chartarum*	IBT 40293	6
	*Trichophyton rubrum*	CBS 118892	2
	*Verruconis gallopava*	CBS 43764	8
Mucoromycota	*Rhizopus microsporus*	ATCC 52814	1
	*Mucor circinelloides*	1006PhL	2

This table lists the clinically relevent fungal species included in our analysis, indicating their taxonomic division, the specific strains examined, and the number of Aqp1 protein homologues identified in each strain.

### Growth of *C. neoformans* aquaglyceroporin mutants in media containing glucose or glycerol as the main carbon source

**Figure 2:**
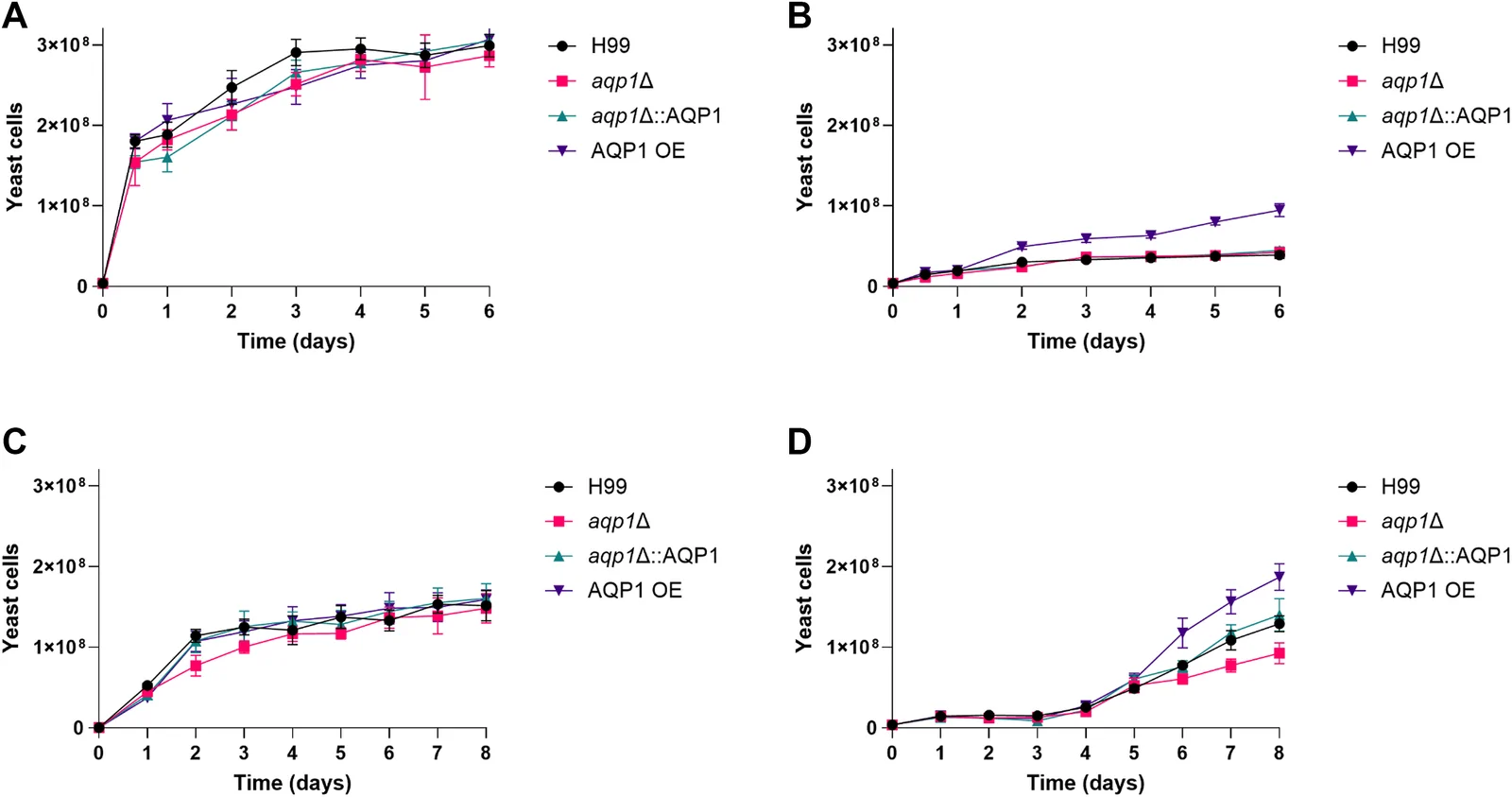
**Comparative growth analysis of *C. neoformans* strains under glycerol as unique carbon source.** The figure shows the growth dynamics of 4 *C. neoformans* strains: H99, *aqp1*Δ, *aqp1*Δ::AQP, and AQP1 OE. Glucose media were used as a control. *A*, growth curves of all strains cultured in yeast nitrogen base (YNB) medium containing 111 mM glucose as the carbon source. Cultures were incubated for 6 days, and cell density was measured every 24 h. *B*, growth curves of all strains grown in YNB medium in which glucose was replaced with 111 mM glycerol as the sole carbon source. *C*, growth curves of the strains incubated in minimal medium (MM) containing 111 mM glucose for 8 days, with cell density measured every 24 h. *D*, growth curves of the strains grown in MM supplemented with 111 mM glycerol instead of glucose, under identical incubation and measurement conditions. Data represents the mean of 3 independent biological replicates. Statistical differences between samples at each data point were evaluated using two-way ANOVA.

Earlier sequence analysis suggested that Aqp1 in *C. neoformans* could act as an aquaglyceroporin capable of transporting glycerol and other small solutes (12). To confirm this experimentally, the wild-type strain H99, the deletion mutant (*aqp1*Δ), the complemented strain (*aqp1*Δ::*AQP1*), and the overexpression strain (*AQP1* OE) were each tested for growth in media where either glucose or glycerol served as the primary carbon source. In YNB media supplemented with glucose, all tested strains showed similar growth rates over 6 days of incubation (Fig. 2*A*), implying that Aqp1 was not required for growth when glucose is available. When glycerol replaced glucose in the same type of medium, only the *AQP1* OE strain manifested an increased growth rate (Fig. 2*B*). This difference was already apparent after 3 days of growth, at which point the *AQP1* OE strain showed a significantly higher growth rate (*p* < 0.0001) than the wild-type strain. Growth in this condition was delayed and modest compared to glucose-grown cultures, yet the overexpression strain continued to proliferate, unlike H99, *aqp1*Δ, and *aqp1*Δ::*AQP1* strains. Growth differences were also assessed in minimal medium (MM) containing either glucose or glycerol as a primary carbon source. In MM–glucose, all tested strains proliferated at similar rates for 8 days (Fig. 2*C*). In the conditions where glycerol was the sole carbon source, a distinct pattern emerged (Fig. 2*D*). Growth was initially slow for all strains, but after about 4 days, the *AQP1* OE strain presented an increase in proliferation rate, reaching the highest cell concentration and showing the steepest increase in growth, followed by H99 and *aqp1*Δ::*AQP1*, which grew at intermediate rates. After 6 days of incubation, the AQP1 OE strain showed a statistically significant (*p* < 0.0001) increase in growth rate compared to the wild-type strain. In contrast, *aqp1*Δ mutant proliferated slowly presenting a statistically (*p* < 0.0262) slower growth rate after 6 days of incubation and remained at the lowest cell concentration during the entire incubation period. These findings indicate that Aqp1 facilitates growth when glycerol is the available carbon source. The superior performance of the *AQP1* OE strain and the impaired growth of the *aqp1*Δ mutant support the view that Aqp1 functions as an aquaglyceroporin in *C. neoformans*, facilitating glycerol uptake and contributing to metabolic flexibility under nutrient-limited conditions.

### Aquaporin Aqp1 does not affect the growth of *C. neoformans* under temperature stress, fluconazole treatment, or melanization

Aquaporins were previously shown to be essential components of high-temperature stress response in various plants, animals, and fungi (9, 40, 41, 42, 43, 44, 45, 46). To determine whether Aqp1 contributes to the thermal stress adaptation of *C. neoformans*, we performed spot dilution assays using the wild-type strain H99, an *aqp1* deletion mutant (*aqp1*Δ), a complemented strain (*aqp1*Δ::*AQP1*), and a strain overexpressing Aqp1 (*AQP1* OE). Ten-fold serial dilutions of each strain were spotted onto YPD agar and incubated at 30, 37, or 39 °C to assess growth under normal and elevated temperature conditions. All strains presented similar growth at each temperature (Fig. S2*A*). To investigate if Aqp1 played a role in low-temperature adaptation, the same set of strains was analyzed on both minimal media (MM) and YPD solid media at 4 °C, and plates were incubated for 6, 12, and 18 days to accommodate slower growth at this temperature (Fig. S2, *B* and *C*). All strains showed similar growth across all tested conditions. This observation suggests that Aqp1 is not required for cellular adaptation to high or low-temperature stress responses in *C. neoformans*. To assess the potential role of aquaporins in antifungal susceptibility, we conducted a dilution spot assay on YPD agar media supplemented with 0, 8, or 12 μg/ml fluconazole. All strains exhibited comparable growth across the tested concentrations (Fig. S2*D*), confirming that aquaporin function does not influence susceptibility or resistance to fluconazole. Additional evaluation of cryptococcal melanin production was performed by spotting wild-type and aquaporin mutant strains onto MM agar plates supplemented with L-DOPA. All tested strains presented a similar rate of melanin production (Fig. S2*E*).

### Aquaporin Aqp1 does not influence cell size, capsule thickness, or buoyancy in nutrient-rich conditions

**Figure 3:**
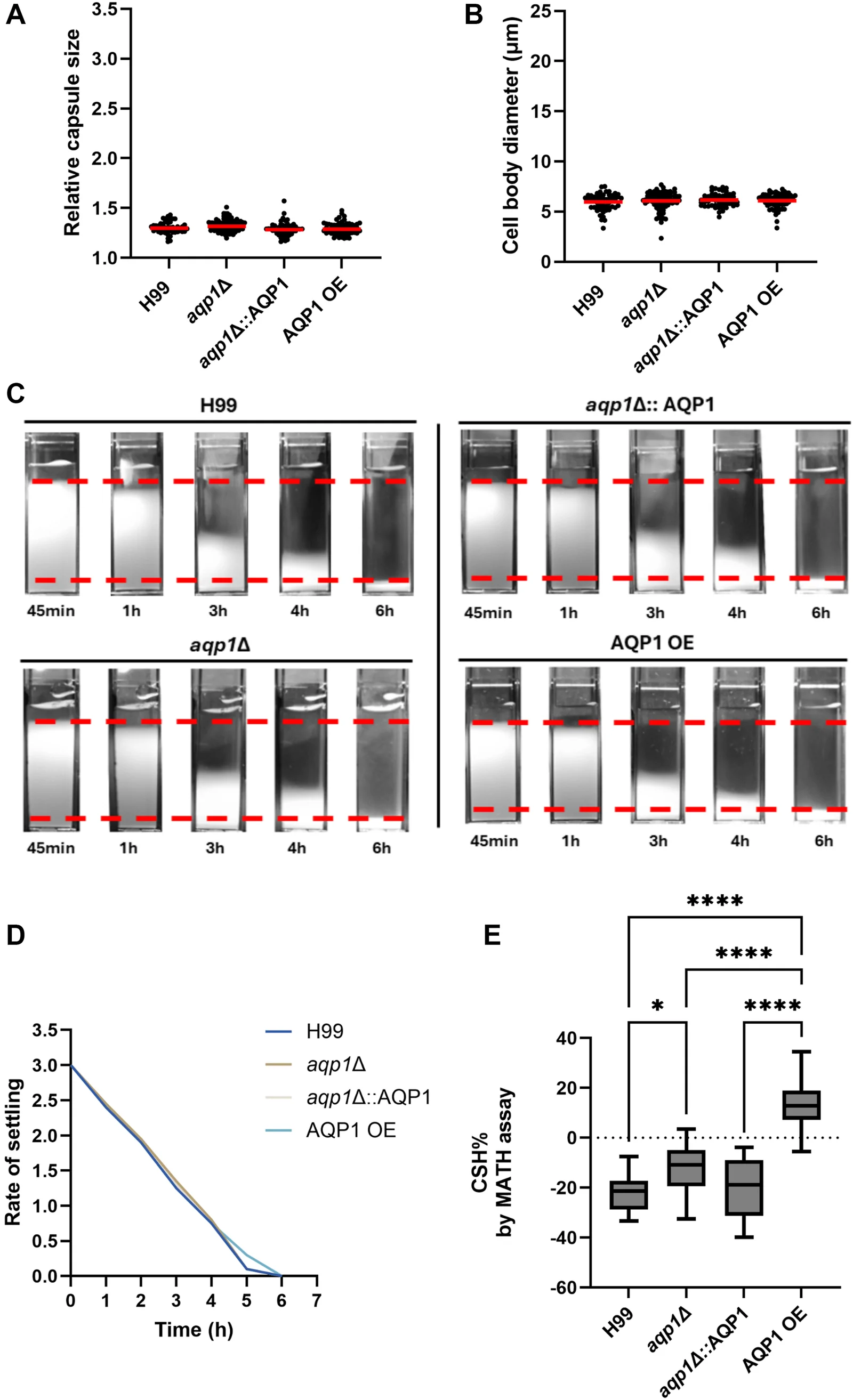
**Analysis of cell buoyancy and hydrophobicity in wild-type, *aqp1*Δ, *aqp1*Δ::*AQP1*, and AQP1 OE strains based on capsule size, cell body diameter, and settling speed.***A*, scatter plot showing the relative capsule size for each strain. *B*, scatter plot displaying cell body diameter measurements. Each point represents an individual cell; bars indicate the mean. *C*, representative images from the settling assay for all 4 strains at various timepoints. *Red dotted lines* indicate the initial and final positions of the cell suspension during settling. *D*, line graph showing the rate of cell settling over time for each strain. (*E*) Box plot presenting the level of hydrophobicity measured for each strain. Statistical analysis was performed using two-way ANOVA test. (∗*p* < 0.05, ∗∗*p* < 0.01, ∗∗∗*p* < 0.001, ∗∗∗*p* < 0.0001).

To examine the potential role of Aqp1 for cryptococcal morphology or cell density, we analyzed cell size, capsule thickness, and buoyancy in wild-type H99, *aqp1*Δ, *aqp1*Δ::*AQP1*, and *AQP1* OE strains following growth in YPD liquid media for 48 h. Quantitative measurements revealed no differences in cell size or capsule dimensions among the tested strains (Fig. 3, *A* and *B*). To assess potential changes in cell density, we used cell buoyancy as a proxy by measuring the rate of passive cell settling in static liquid cultures, under the assumption that cells with lower density would exhibit more buoyancy. All strains displayed similar buoyancy, with no differences in settling rate between strains (Fig. 3, *C* and *D*). Thus, Aqp1 did not significantly alter cell size, capsular size, or buoyancy of *C. neoformans* cells under nutrient-rich growth conditions. The assessment of the changes in cell surface hydrophobicity was evaluated using the MATH assay, which revealed that the overexpression mutant strain presented a substantial increase in cell surface hydrophobicity (Fig. 3*E*). Additionally, the deletion mutant strain presented a small but significant change in cell surface hydrophobicity, supporting previous suggestions that aquaporin may play a crucial role in processes managing cellular hydrophobicity (12).

### Aquaporin overexpression enhances capsule and cell enlargement under capsule-inducing conditions

**Figure 4:**
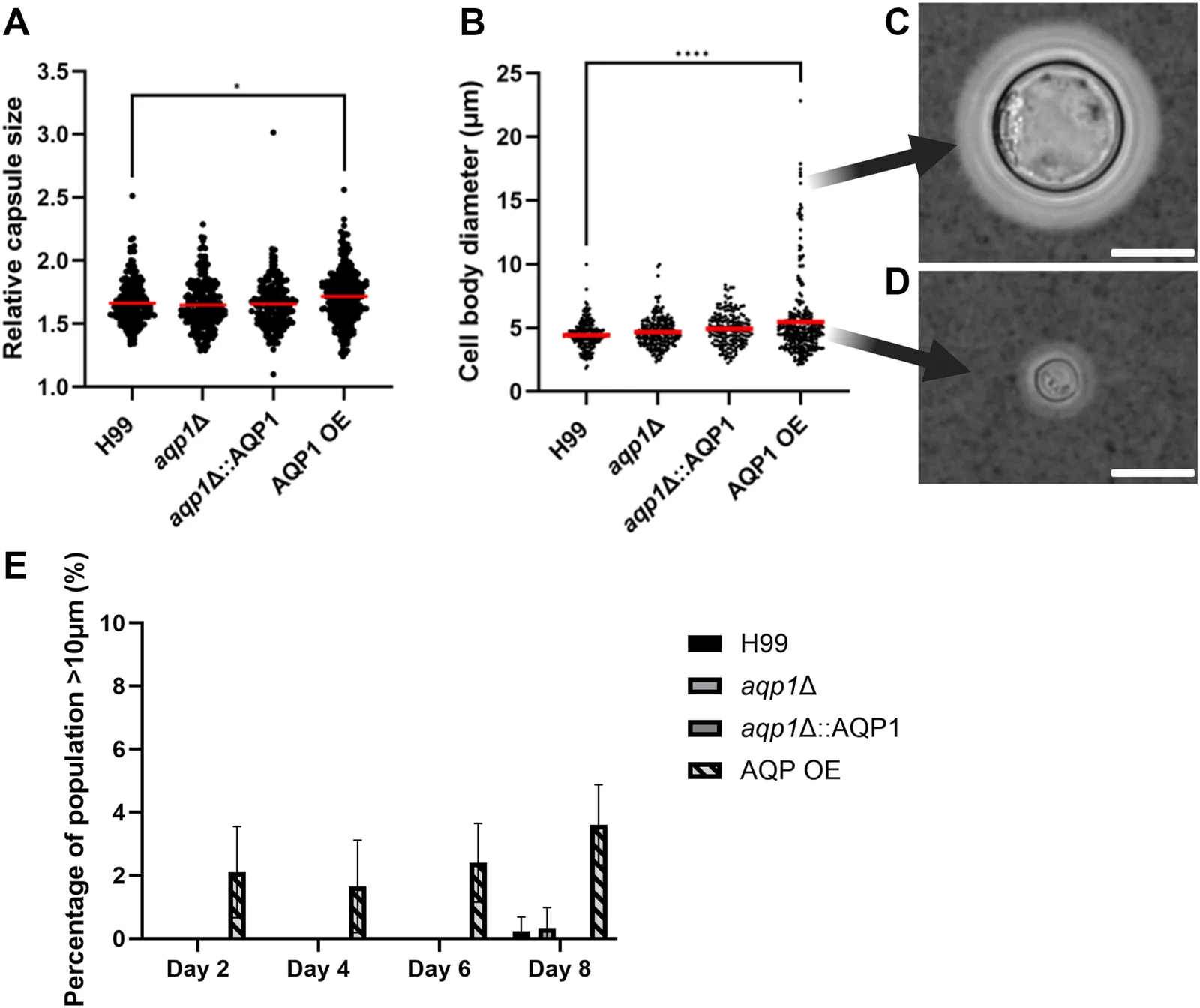
**Analysis of polysaccharide capsule size and cell body diameter in wild-type, *AQP1* deletion, complemented, and overexpression strains grown in capsule-inducing minimal media.***A*, scatter plot showing relative capsule size for all 4 strains. *B*, scatter plot displaying cell body diameter measurements across the same strains. Each point represents an individual cell; bars indicate mean ± SD. *C*, India ink-stained image of an enlarged cell observed within the AQP1-overexpressing strain population, captured under a 40× objective. *D*, India ink-stained image of a representative cell with average size, also imaged under a 40× objective. Scale bar represents 10 μm. Panels *C* and *D* highlight morphological heterogeneity observed in the AQP1-overexpressing strain. *E*, Bar graph representing the mean percentage of cell population exceeding 10 μm in diameter measured after 2, 4, 6, and 8 days of incubation in MM at 30 °C. Data represent the mean from 3 independent biological replicates. Statistical analysis was performed using two-way ANOVA test(∗*p* < 0.05, ∗∗*p* < 0.01, ∗∗∗*p* < 0.001, ∗∗∗*p* < 0.0001).

The cellular morphology of *C. neoformans* is strongly influenced by the surrounding environmental factors and various stress conditions that may modulate its adaptive stress responses (26, 28, 31, 47, 48, 49). To evaluate if Aqp1 could modulate cell morphology in capsule-inducing conditions, we cultured wild-type and aquaporin mutant strains in MM at 30 °C for 3 days. Under these conditions, the *AQP1*-overexpressing cells showed increased average cell size and capsule thickness compared to the other tested strains (Fig. 4, *A* and *B*). Surprisingly, a substantial subpopulation of cells from the aquaporin overexpression strain exceeded 10 μm in diameter, a phenotype that was not observed in the wild-type or mutant strains (Fig. 4, *C* and *D*). Further evaluation of the cell size of tested strains for an extended period (up to 8 days) showed that a small subpopulation of cells in the strain with overexpressed aquaporin 1 exceeded 10 μm (Fig. 4*E*). Flow cytometry of cells stained with propidium iodide (PI) indicated increased DNA content in AQP1 OE strain (Fig. S3). These findings suggest that elevated Aqp1 expression strongly promotes cellular and capsular enlargement in a small subpopulation of cells exposed to low-nutrient, capsule-inducing conditions.

### Aqp1 influences cell size in low-inoculum cultures grown under capsule-inducing conditions

**Figure 5:**
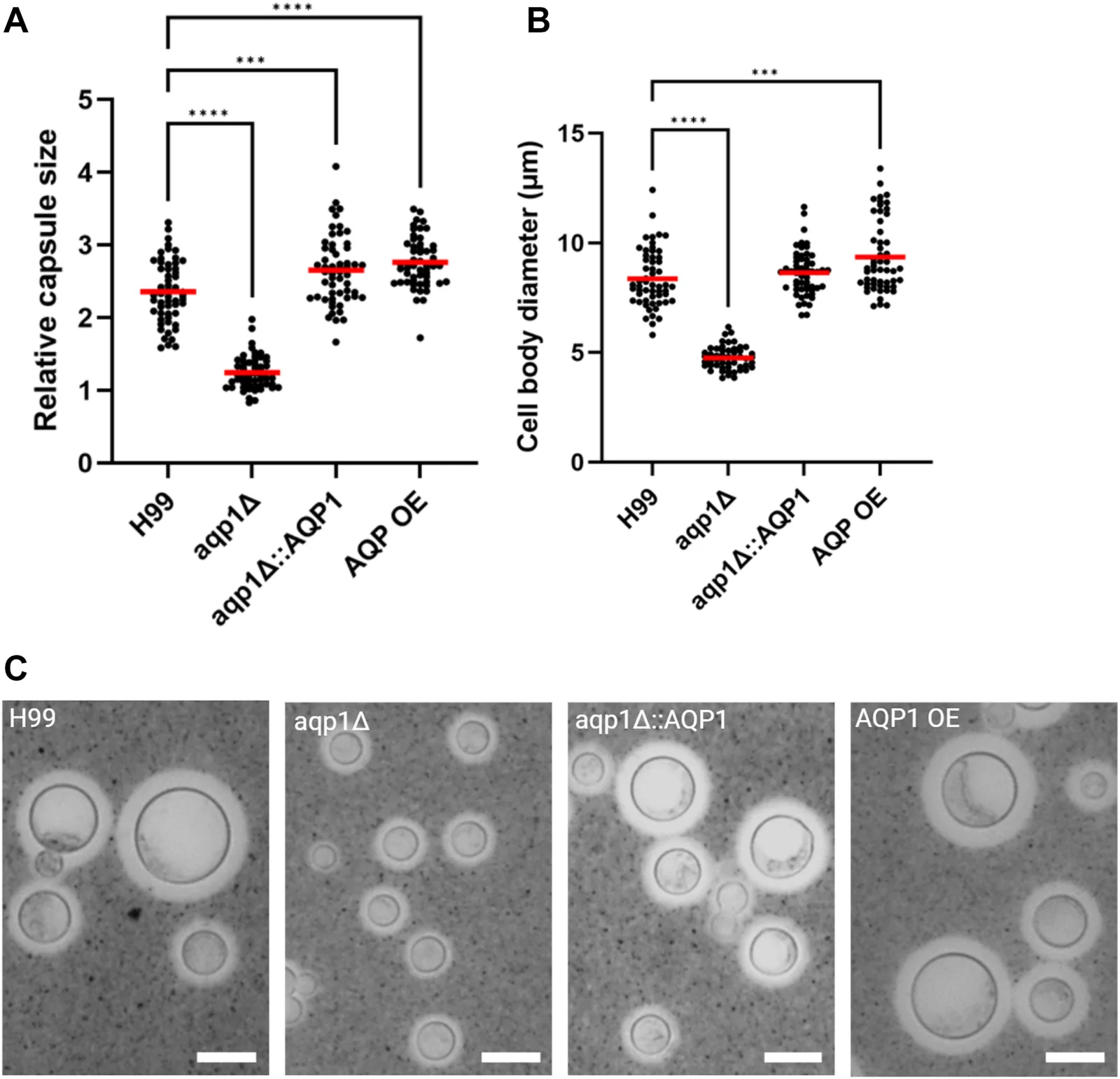
**Analysis of cellular morphology in wild-type, AQP1 deletion, complemented, and overexpression strains grown from low inoculum in minimal medium.***A*, scatter plot showing relative capsule size across the 4 strains. *B*, scatter plot showing cell body diameter measurements for the same strains. Each point represents an individual cell; bars indicate the mean. *C*, representative India ink-stained images of each strain captured under a 40× objective, illustrating differences in capsule and cell body size. The scale bar represents 10 μm. Data represent the mean from 3 independent biological replicates. Statistical analysis was performed using a two-way ANOVA test (∗*p* < 0.05, ∗∗*p* < 0.01, ∗∗∗*p* < 0.001, ∗∗∗*p* < 0.0001).

To further explore the role of Aqp1 in cryptococcal morphogenesis under capsule-inducing conditions, we inoculated wild-type and aquaporin mutant strains into minimal medium at a low starting density (1000 cells per mL of culture) and incubated them at 30 °C for 4 days. Cell body and capsule sizes were then assessed by India ink staining and light microscopy. The *AQP1*-overexpressing strain formed cells with modest but statistically significant increases in capsule thickness compared to the wild-type strain (Fig. 5*A*). Additionally, the *aqp1*Δ strain displayed a significantly reduced average cell body diameter (∼5 μm), while WT, complemented, and overexpression strains showed a marked increase in cell size (Fig. 5*B*). In these conditions, the aquaporin deletion mutant presented a uniform cell morphology without the presence of large Titan-like cells (Fig. 5*C*). These results indicate that Aqp1 contributes to cell body expansion in response to low-inoculum growth in capsule-inducing conditions, with its absence limiting size and its overexpression enhancing cellular and capsular size.

### Aqp1-mediated cell enlargement is pH-dependent but uncoupled from growth rate changes in *C. neoformans*

**Figure 6:**
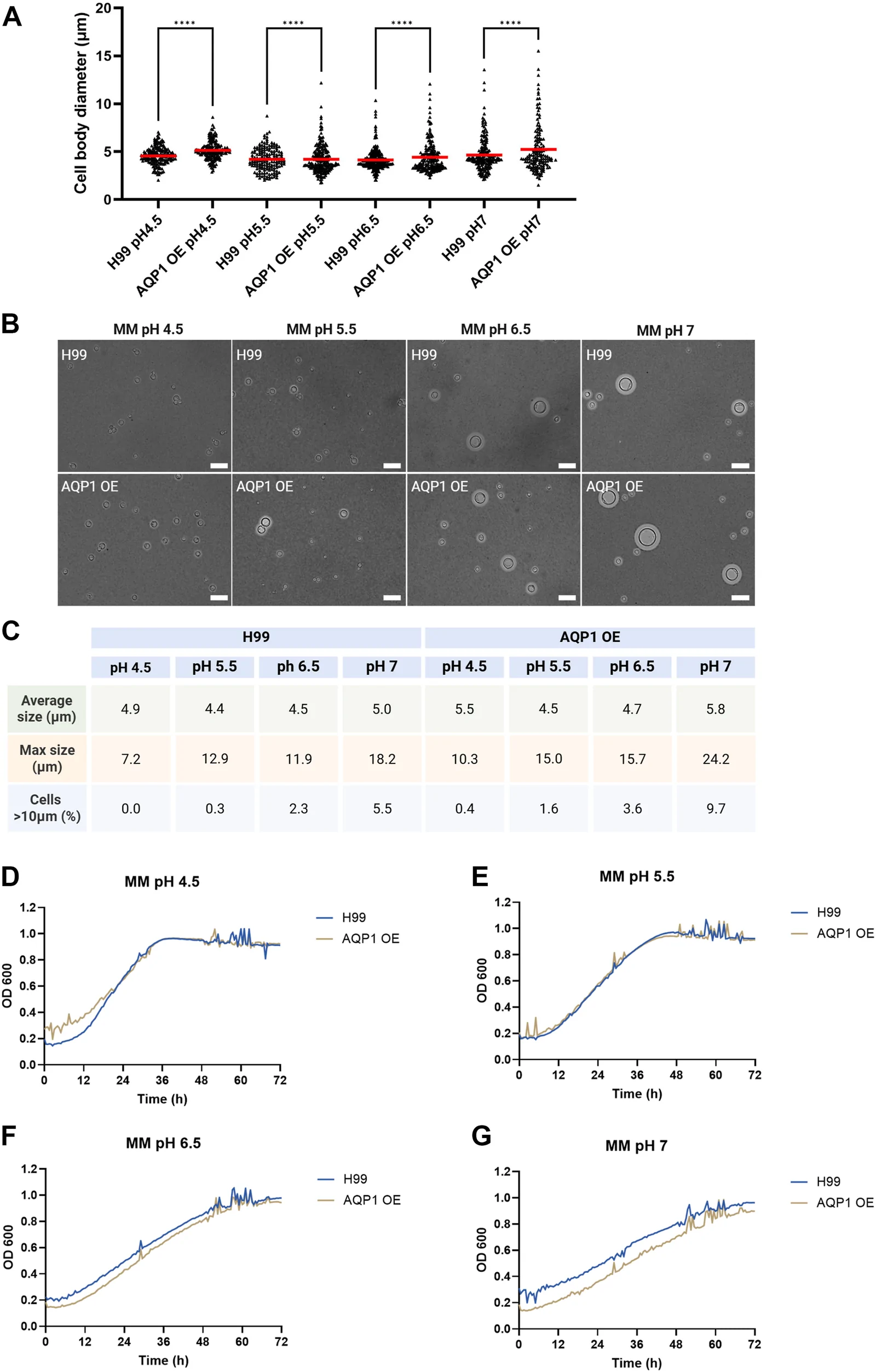
**Effect of pH on cell morphology and growth dynamics in wild-type and AQP1-overexpressing (AQP1 OE) strains.***A*, scatter plot showing cell body diameter of wild-type (H99) and *AQP1* OE strains grown in minimal medium at pH 4.5, 5.5, 6.5, and 7.0. Each point represents an individual cell; *red bars* indicate the mean. *B*, representative India ink–stained images captured under a 40× objective for each strain and pH condition, illustrating morphological differences. *C*, summary table presenting the average and maximal cell body diameter for both strains at each pH, along with the percentage of the cell population exceeding 10 μm in diameter. Growth curves of wild-type and AQP1 OE strains in minimal medium adjusted to pH 4.5 (*D*), 5.5 (*E*), 6.5 (*F*), and 7.0 (*G*). Optical density at 600 nm was measured over time to assess growth dynamics. Data in panels *A*, *D*–*G* represent the mean from 3 independent biological replicates. The scale bar represents 10 μm. Statistical analysis was performed using a two-way ANOVA test (∗*p* < 0.05, ∗∗*p* < 0.01, ∗∗∗*p* < 0.001, ∗∗∗*p* < 0.0001).

A standard minimal medium used to induce the production of cryptococcal polysaccharide capsule was adjusted to acidic pH 5.5. To evaluate if Aqp1-associated cell enlargement could be influenced by extracellular pH, we cultured the wild-type and *AQP1*-overexpressing strains in MM adjusted to pH 4.5, 5.5, 6.5, or neutral pH 7. After 3 days of incubation at 30 °C, cells were suspended in India ink and analyzed by light microscopy to quantify cell size. At all tested pH levels, the *AQP1*-overexpressing mutant produced significantly larger cells than the wild-type strain (Fig. 6, *A* and *B*). However, maximal cell sizes in both strains decreased as pH decreased. Specifically, in media at pH 4.5, the largest observed cell diameters were 7.2 μm for wild-type and 10.3 μm for *AQP1* OE, whereas at pH 7.0 they reached 18.2 μm and 24.2 μm for wild-type and aquaporin-overexpressing mutant strains, respectively. Additionally, the proportion of cells exceeding 10 μm in diameter increased with pH in both strains, ranging from 0% at pH 4.5 to 5.5% (WT) and 9.7% (*AQP1* OE) at pH 7 (Fig. 6*C*). These findings suggest that Aqp1-mediated cell enlargement is pH-sensitive, with more pronounced effects at neutral pH.

After observing that cellular size was pH-dependent and mediated by aquaporin expression levels, we analyzed whether AQP1 could influence cellular growth rate under varying pH conditions. In this experiment, we compared the growth curves of wild-type and *AQP1*-overexpressing strains in minimal media adjusted to pH 4.5, 5.5, 6.5, and 7.0. No measurable differences in growth rates were observed between the two strains at pH 4.5, 5.5, or 6.5 (Fig. 6, *D*–*F*). At pH 7.0, the wild-type strain proliferation rate was slightly faster than in the case of the overexpressing mutant strain; however, the difference was minimal and unlikely to be biologically significant (Fig. 6*G*). These results indicate that *AQP1* overexpression did not substantially impact growth under standard minimal media conditions across a range of different pH values. However, increasing the pH of the MM had a pronounced adverse effect on overall growth, greatly extending the time required for cultures to reach the plateau phase, suggesting that growth was slowed under less acidic conditions.

### Aquaporin modulated ion content in *C. neoformans*

**Figure 7:**
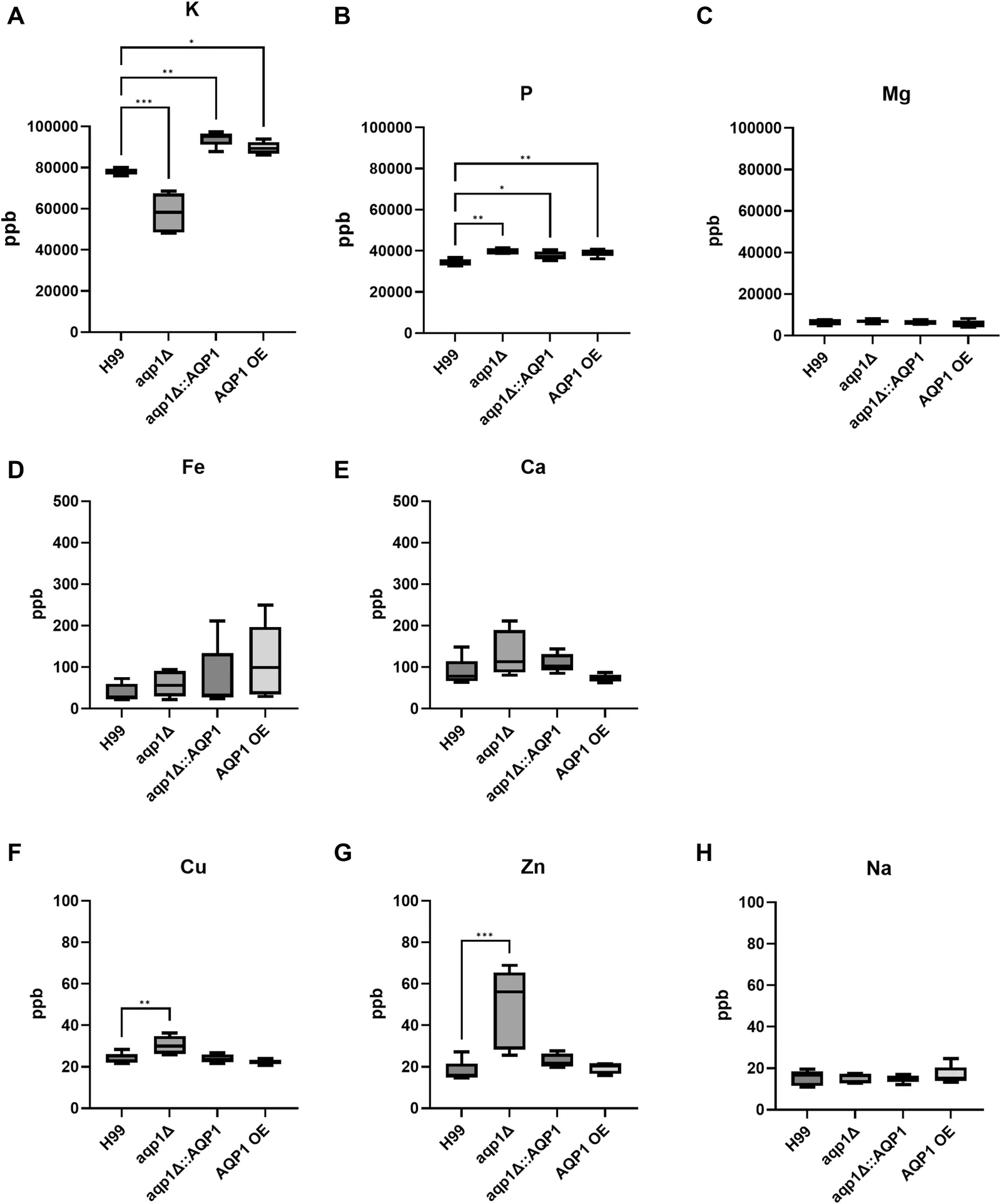
**Inductively Coupled Plasma Mass Spectrometry (ICP-MS) analysis of selected elemental concentrations in wild-type, AQP1 deletion, complemented, and overexpression strains grown in standard minimal media.***Box plots* represent the mean from 5 biological replicates. Elements analyzed include (*A*) potassium (K), (*B*) phosphorus (P), (*C*) magnesium (Mg), (*D*) iron (Fe), (*E*) calcium (Ca), (*F*) copper (Cu), (*G*) zinc (Zn), and (*H*) sodium (Na). Elemental concentrations were assessed to evaluate potential roles of AQP1 in ion homeostasis and transport. Data represents the mean of 5 independent biological replicates. Statistical analysis was performed using two-way ANOVA test (∗*p* < 0.05, ∗∗*p* < 0.01, ∗∗∗*p* < 0.001, ∗∗∗*p* < 0.0001).

**Figure 8:**
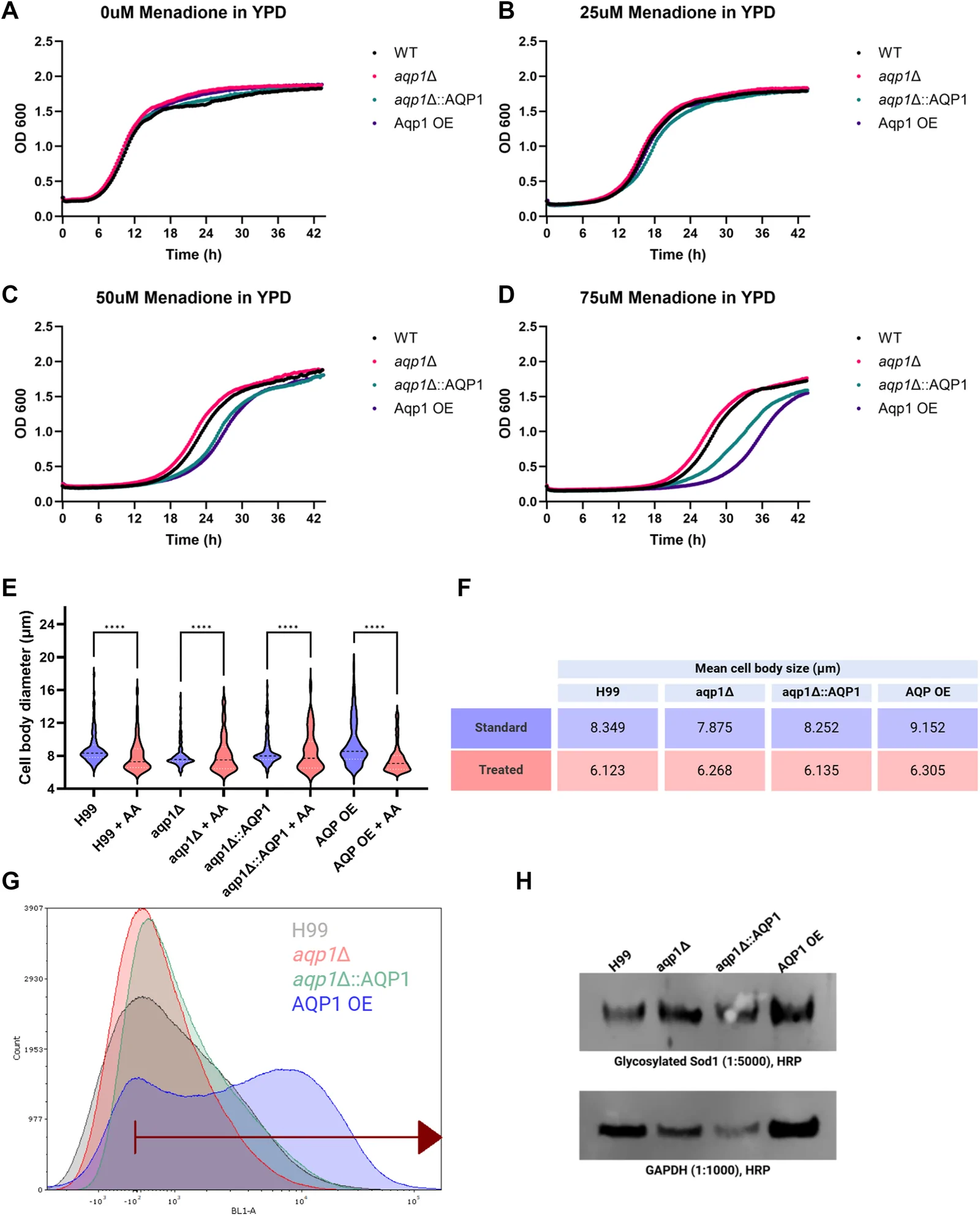
**Impact of ROS and antioxidant treatment on growth and cell morphology in *Cryptococcus neoformans*.** Growth dynamics and cell size distribution were analyzed in 4 *C. neoformans* strains: wild-type H99, *aqp1*Δ, *aqp1*Δ::*AQP1*, and *AQP1* OE. *PanelsA–D* show growth curves in YPD medium under increasing oxidative stress induced by menadione: (*A*) control (YPD only); (*B*) YPD + 25 μM menadione (*C*) YPD + 50 μM menadione; (*D*) YPD + 75 μM menadione. *E*, violin plots of cell body sizes (>6 μm diameter) under titan cell-inducing conditions, comparing untreated and 3 mM ascorbic acid (AA)-treated cultures. Statistical differences between samples were evaluated using two-way ANOVA. (∗*p* < 0.05, ∗∗*p* < 0.01, ∗∗∗*p* < 0.001, ∗∗∗*p* < 0.0001). *F*, table summarizing the data from panel *E*, showing average cell sizes for each strain with and without AA treatment. *G*, Quantification of intracellular ROS levels by flow cytometry following incubation of cells with 20 μM H_2_DCFDA fluorescent probe. *H*, Sod1 protein abundance was evaluated by Western blot analysis in wild-type H99, *aqp1Δ*, *aqp1Δ::AQP1*, and *AQP1* overexpression strains. GAPDH was used as a loading control.

To explore the involvement of *AQP1* in maintaining ion balance in *C. neoformans* cells, we utilized Inductively Coupled Plasma Mass Spectrometry (ICP-MS) on wild-type, *aqp1*Δ, complemented, and overexpression strains grown in MM. (Fig. 7). The aquaporin deletion mutant presented a significantly lower (*p* < 0.001) intracellular potassium (K) concentration than the wild type (Fig. 7*A*). In contrast, both the complemented and overexpression strains showed slightly elevated K levels compared with the wild-type strain. All 3 mutant strains displayed slightly higher yet statistically significant intracellular phosphorus (P) concentrations than the WT (Fig. 8*B*). Notably, the deletion mutant presented substantially elevated levels of copper (Cu) and zinc (Zn) compared to wild type, suggesting a potential role for *AQP1* in maintaining metal ion balance (Fig. 7, *F* and *G*). These data indicate a link between *AQP1* expression and the regulation of specific intracellular element concentrations. Given that the *AQP1* deletion mutant showed significantly elevated intracellular levels of copper (Cu) and zinc (Zn) compared to the wild-type strain, we hypothesized that *AQP1* may influence the function of copper- and zinc-dependent enzymes such as superoxide dismutase 1 (*SOD1*). We thus examined Cu and Zn tolerance in H99 and aquaporin mutant strains. The *AQP1* OE strain grew slightly better at 0.312 mM CuSO_4_ at 24 h, but by 48 to 72 h, all strains showed similar growth at 0.625 mM CuSO_4_ (Fig. S3, *A*–*C*). Zinc tolerance was comparable across strains, with growth at 1.25 mM ZnSO_4_ after 48 to 72 h (Fig. S3, *D*–*F*). These results indicate that aquaporin expression has little effect on metal resistance in *C. neoformans*.

### Cryptococcal aquaporin modulates growth linked to intracellular ROS homeostasis

To examine if *AQP1* plays a role in the oxidative stress response, we examined growth dynamics and cell morphology under conditions of reactive oxygen species (ROS) stress and antioxidant treatment (Fig. 8). Growth curves were generated for WT, *and a set* of aquaporin mutant strains cultured in YPD medium with increasing concentrations (0, 25, 50, and 75 μM) of menadione (vitamin K3), which promotes oxidative damage. While all strains grew similarly under control conditions (0 μM), the *AQP1*-overexpressing strain exhibited a reduced growth rate relative to the wild-type and deletion strains when exposed to 50 and 75 μM menadione (Fig. 8, *A*–*D*), suggesting that *AQP1* overexpression increased sensitivity to ROS stress. Visualization of intracellular ROS levels revealed subtle differences in fluorescent signal intensity among the tested strains (Fig. S5). The wild-type, *aqp1*Δ mutant, and complemented strains showed comparable levels of intracellular staining, whereas the *AQP1* overexpression strain exhibited a consistently brighter fluorescence profile. Notably, when the strains were treated with the antioxidant ascorbic acid (AA), they displayed significantly reduced cell size compared with untreated cells (Fig. 8, *E* and *F*). Furthermore, the quantitative measurement of intracellular ROS using flow cytometry coupled with H2DCFDA (2′,7′-dichlorodihydrofluorescein diacetate) confirmed a substantial accumulation of ROS in the AQP1 overexpression mutant strain, indicating greater ROS accumulation within the cells (Fig. 8*G*). Western blot analysis of intracellular Sod1 indicated no difference in Sod1 presence between all tested strains. These differences further support the idea that *AQP1* overexpression leads to accumulation of intracellular ROS levels in *C. neoformans* cells.

### Aqp1 localizes to intracellular membranes and modulates titan cell formation and vacuole morphology during *In vitro* induction

**Figure 9:**
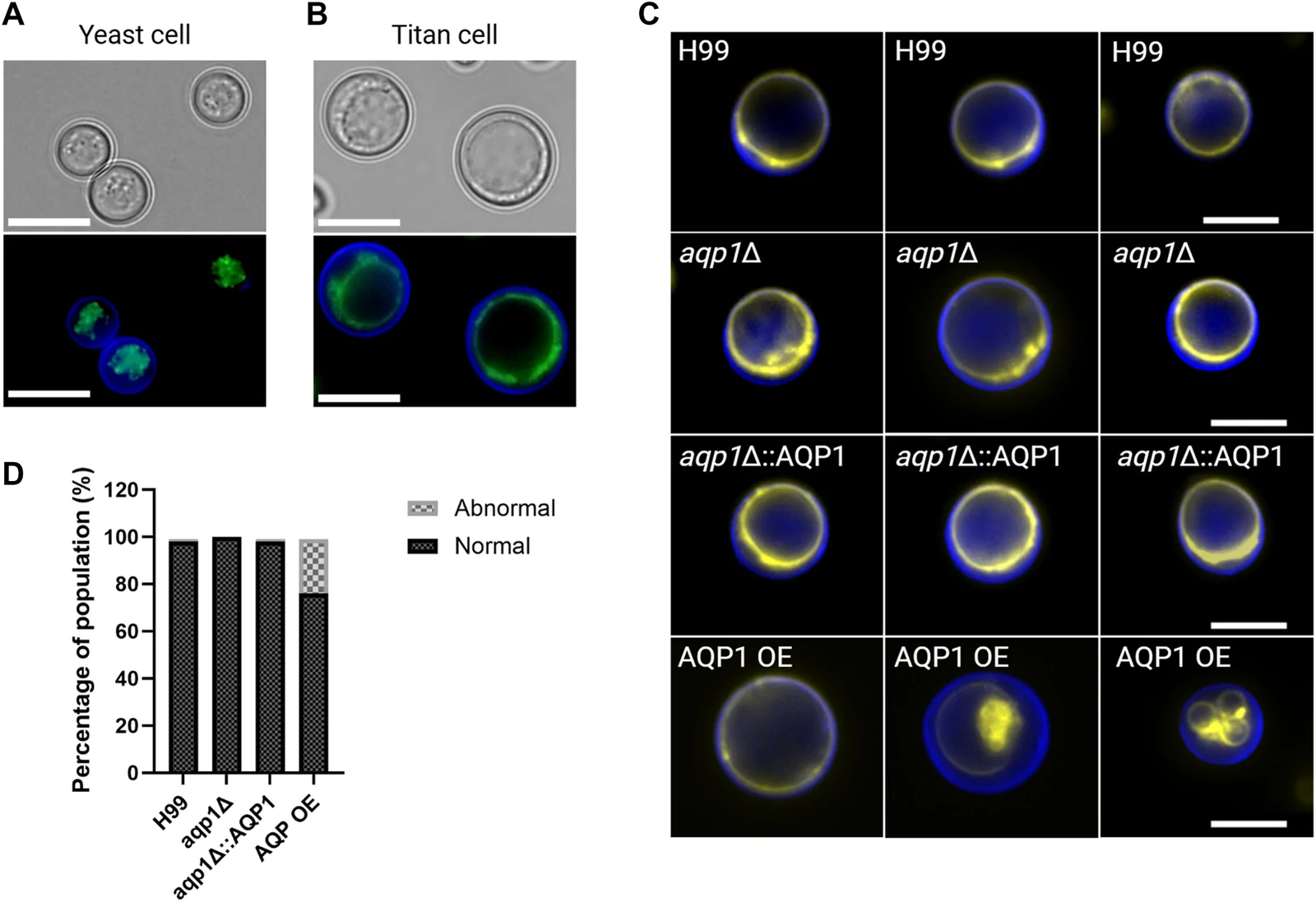
**Localization of Aqp1 and analysis of vacuole morphology in yeast and titan-like cells.***A*, Differential interference contrast (DIC) and fluorescence images of yeast-form *C. neoformans* cells expressing Aqp1-GFP. *Green* fluorescence indicates Aqp1-GFP localization, and the cell wall was stained with Uvitex 2B (*blue*). *B*, DIC and fluorescence images of titan-like cells expressing Aqp1-GFP under titan cell–inducing conditions. *Green* fluorescence indicates the localization of GFP-tagged aquaporin in titan-like cells, and the cell wall was stained with Uvitex 2B (*blue*). *C*, analysis of vacuole morphology in titan-like cells of WT, *aqp1*Δ, *aqp1*Δ::AQP1, and AQP1 OE strains. Vacuoles were stained with FM4-64 (*yellow*), and cell walls were stained with Uvitex 2B (*blue*). Scale bar represents 10 μm. *D*, the stacked column graph visualizes the data for the percentage of titan cell population found with an abnormal phenotype of the large central vacuole (<70 cells).

As reported in previous research, aquaporin 1 localizes to the internal membrane in yeast cells of *C. neoformans* (Fig. 9*A*) (12). Visualization of titan-like cells by fluorescent microscopy revealed aquaporin localization at the peripheral membrane, in the cytoplasmic compartment surrounding the large central vacuole (Fig. 9*B*).

Given our earlier observations of frequent cell enlargement in the *AQP1*-overexpressing strain, we investigated whether aquaporin 1 influenced the intracellular morphology of titan cells in *C. neoformans*. One of the significant characteristics of cryptococcal titan cells is their large central vacuole (31, 33). Vacuolar FM4-64 staining revealed striking vacuolar abnormalities in vacuolar morphology in the *AQP1*-overexpressing strain, affecting approximately 24% of titan-like enlarged cells (Fig. 9*C*). In contrast, the frequency of vacuolar defects in titan-like cells of the wild-type, *aqp1*Δ, and complemented strains remained close to 0%. Together, these findings suggest that Aqp1 facilitates titan cell enlargement and that its overexpression may disrupt vacuole homeostasis in titan-like cells.

## Discussion

Aquaporins are integral membrane proteins known for functioning to facilitate water transport across biological membranes, yet their roles in fungal pathogens, particularly *C. neoformans*, remain incompletely understood. Prior to this study, Aqp1 in *C. neoformans* was known to be transcriptionally induced by osmotic and oxidative stress *via* the HOG pathway yet was largely dispensable for adaptation to environmental stressors and virulence (12). Aqp1 was shown to play a minor role in regulating cell-surface hydrophobicity and to be involved in maintaining metabolic homeostasis (12). Deletion of *AQP1* led to the accumulation of various metabolites and enhanced competitive survival, while overexpression of *AQP1* reduced metabolite levels (12). Here, we initially sought to evaluate potential contribution of aquaporin in the process of thermal stress response and to test if aquaporin channels played an important role in the regulation of cellular buoyancy. In this study, we investigated the contribution of the *C. neoformans* aquaporin Aqp1 to growth, morphogenesis, and titan cell formation under various environmental conditions. Our results reveal that Aqp1 does not influence cryptococcal growth under standard, nutrient-rich laboratory culture conditions, but significantly impacts cell enlargement and morphological adaptation in host-like environments that confer cellular stress and may additionally have a role in protection against heavy metals.

The growth analyses presented here support a role for Aqp1 in glycerol utilization by *C. neoformans*. Strains expressing Aqp1 grew normally in media containing glucose, indicating that this protein was not required for metabolism of the preferred carbon source. However, clear differences emerged when glycerol was provided as the main carbon source. Under those conditions, cells lacking *AQP1* showed reduced proliferation, while cells overexpressing the gene showed increased growth. These findings support previous predictions that Aqp1 acts as an aquaglyceroporin, facilitating the movement of glycerol across the cell membrane. Initial phenotypic analyses using spot assays demonstrated that neither deletion nor overexpression of *AQP1* altered growth under thermal stress (30 °C, 37 °C, or 39 °C) or during prolonged incubation at low temperatures (4 °C). Elimination or overexpression of aquaporin in *C. neoformans* had no visible effect on its susceptibility to fluconazole treatment. Similarly, Aqp1 did not affect cell size, capsule thickness, or buoyancy in nutrient-rich medium, suggesting that under non-capsule-inducing conditions, Aqp1 was dispensable for basic cellular physiology. In contrast, under capsule-inducing conditions, particularly in minimal medium, Aqp1 overexpression resulted in significantly increased capsule size and cell body diameter, with a notable subpopulation of cells exceeding 10 μm in diameter—reminiscent of titan cells (32, 33, 34, 50, 51, 52). This phenotype was further accentuated when cells were cultured under low-density conditions, which are known to favor morphological diversification. The *aqp1* deletion mutant exhibited significantly reduced cell size, whereas overexpression of Aqp1 maintained an enlarged phenotype, supporting a role for Aqp1 in promoting cellular expansion. Since pH can modulate fungal physiology, including capsule formation and cell size, we investigated whether Aqp1-mediated cell enlargement is correlated with a different spectrum of pH (53, 54, 55). Our findings show that less acidic pH conditions promoted the emergence of enlarged and titan-like cells in both wild-type and *AQP1*-overexpressing strains, with a pH-dependent increase in the proportion of cells >10 μm, which is consistent with previous observations suggesting the importance of neutral pH on titan cells formation (31). Interestingly, the increase in pH not only favored the emergence of enlarged and titan-like cells but also correlated with a slower overall growth rate, independently of *AQP1* expression. Cultures grown at higher, less acidic pH levels exhibited delayed progression to the plateau phase, suggesting that more neutral conditions may impose physiological constraints that decelerate proliferation while promoting morphological remodeling.

Using established *in vitro* titan cell induction media, we tested the role of Aqp1 in titan cell formation. Titan cells are critical for the virulence of *C. neoformans* because their enlarged size makes them resistant to phagocytosis by host immune cells, enhancing survival within the host (33, 56). Formation of the titan cell during the infection may contribute to immune modulation and promote dissemination by generating smaller progeny cells (27, 33, 35). The overexpression of *AQP1* led to a significant increase in average cell size and frequency of titan-like cells, whereas the *aqp1*Δ mutant failed to undergo typical size enlargement.

Our findings show that Aqp1 is involved in intracellular ionic, oxidative, and morphological homeostasis in *C. neoformans*. The deletion mutant showed significantly reduced intracellular levels of potassium, copper, and zinc, indicating that Aqp1 can influence ion balance. These findings suggest that cryptococcal aquaporin may play a key role in metal distribution and metabolism, comparable to the role of mammalian Aqp4 in the brain (57). The elevated Cu and Zn levels are particularly notable given their essential role as cofactors for superoxide dismutase 1 (Sod1), a major antioxidant enzyme that protects against various forms of reactive oxygen species. Both metals are also toxic to fungi at higher concentrations (58, 59, 60). To explore this connection, we analyzed the response of *AQP1*-altered strains to oxidative stress and found that *AQP1* overexpression increased sensitivity to ROS, while its deletion had a modest protective effect. Additionally, titan cell formation, which is linked to virulence and immune evasion, was modulated not only by ROS levels but also by *AQP1* expression (61). The *AQP1*-overexpressing strain exhibited the largest titan-like cells, while the *aqp1*Δ strain formed a population of significantly smaller cells. In addition, antioxidant supplementation suppressed cell enlargement, confirming previously observed roles for oxidative signaling in titan morphogenesis (61). Interestingly, although the *AQP1*-overexpressing strain exhibited a significant increase in intracellular ROS levels, the intracellular abundance of Sod1 remained comparable among all tested strains, suggesting that the elevated ROS accumulation observed upon Aqp1 overexpression is not associated with altered Sod1 expression. These data support a model in which Aqp1 influences not only water and solute flux but also intracellular redox balance, potentially through its effects on metal ion availability and Sod1 activity, thereby shaping the pathogen’s stress response and morphological adaptation. One defining feature of cryptococcal titan cells is the presence of a large vacuole, which is involved in maintaining intracellular organization and turgor pressure (31, 33, 62, 63). In our study, *AQP1* overexpression led to frequent vacuolar abnormalities in titan-like cells, a phenotype rarely seen in wild-type or control strains. This suggests that while proper activity of Aqp1 promotes cell enlargement, its overexpression may disrupt vacuole integrity, possibly because excessive water influx overwhelms the cell’s ability to maintain appropriate vacuolar morphology.

Taken together, our findings establish a novel role for the aquaporin in regulating cell enlargement, stress adaptation, and virulence factor expression in *C*. *neoformans*. While it is not essential for growth under optimal conditions, Aqp1 may be a key factor for navigating morphogenetic responses to host-like stimuli such as low nutrient availability, pH variation, or oxidative stress. In this study, we demonstrate that cryptococcal Aqp1 influences intracellular ion composition, including levels of elements essential for antioxidant defense. Its presence can modulate the intracellular response to ROS, which in turn can affect the formation of titan cells (61). Finally, *AQP1* overexpression leads to vacuolar abnormalities in titan-like cells, suggesting that unbalanced water influx may compromise intracellular organization during enhanced cell expansion.

These findings provide new insights into the molecular function of fungal aquaporin with a specific focus on ROS-dependent morphogenesis of cryptococcal titan cells. Future studies should analyze the molecular pathways connecting Aqp1 to stress signaling and explore its potential as a target for novel antifungal drugs.

## Experimental procedures

### Strains and media

Wild-type *C. neoformans* strain H99 and all aquaporin deletion, complemented strain, and overexpression mutants were obtained from the Yong-Sun Bahn Laboratory (Table S1) at Yonsei University (South Korea) (12). All strains were routinely cultured in YPD liquid or solid medium (BD Difco) at 30 °C.

### Structural modeling and functional prediction

The similarity of the amino acid primary sequence of cryptococcal Aqp1 (CNAG_01742) was compared to human aquaporins using BLASTp. To gain insight into the structural characteristics of the cryptococcal aquaporin Aqp1, we performed 3D protein structure prediction using AlphaFold3 (64) A similar prediction structural analysis was made for Aqp1 homologs present in other *C. gattii*, *Malassezia furfur*, *Candidozyma auris*, *C. parapsilosis*, and *Mucor circinelloides*. AlphaFold structures of cryptococcal Aqp1 and human AQP1 and AQP8 were structurally aligned with a Smith-Waterman algorithm using UCSF ChimeraX version 1.9 (65). The DeepTMHMM topology prediction model and SignalP 6.0 signal peptide prediction algorithm were used to evaluate the additional C-terminal domain of the cryptococcal Aqp1 (65, 66). The search for aquaporin domain (PF00230) containing proteins in other clinically relevant fungal species was performed *via* OrthoMCL DB (67).

### Serial spot dilution assay for thermal and fluconazole sensitivity

Overnight YPD cultures were washed with DPBS, adjusted to a final concentration of 10^4^ cells in 3 μl, and used for a 10-fold serial dilution. Cells were spotted onto solid media in 3 μl spots, and the plates were incubated at the respective temperature and photographed. To assess the melanization of cryptococcal cells, overnight cultures were washed with DPBS, adjusted to a final concentration of 10^4^. A total of 3 μl of each culture were spotted onto MM agar media supplemented with L-DOPA and incubated for 3 days at 30 °C.

### Glycerol-based growth analysis

Yeast Nitrogen Base w/o amino acids and ammonium sulfate (YNB) (Difco) medium was prepared by dissolving 1.7 g of YNB base and 40 mM (NH_4_)_2_SO_4_ (Sigma) in 1 L of water and supplementing with 111 mM of the indicated carbon source (glucose or glycerol). The pH was adjusted to 5.5 before sterilization with a vacuum filtration system Stericup (MilliporeSigma). Minimal media (MM) was prepared with 111 mM of carbon source (glucose or glycerol), 13 mM glycine (Sigma), 10 mM MgSO_4_ (Sigma), 29.4 mM KH_2_PO_4_ (Sigma), 3 μM thiamine-HCl, and adjusted to pH 5.5. A single colony of each strain was used to inoculate 4 ml of YPD media in 14 ml bottom-round tubes (Falcon), for 2days with agitation at 30 °C. Yeast cells were harvested, washed 2 times with DPBS, and counted with a hemocytometer. Subsequently, 1 × 10^6^ cells/ml were inoculated into 14 ml bottom-round tubes containing 4 ml of YNB or MM media with glycerol or glucose as the sole carbon source and incubated with agitation at 30 °C. Glucose media were used as a control. The cells were counted daily throughout the experiment. Results represent the cell density in the entire sample. Growth data were visualized and analyzed using GraphPad Prism 10.1.1.

### Cell morphology, buoyancy, and hydrophobicity assay

Cell cultures were incubated in YPD liquid medium for 2 days at 30 °C and washed with DPBS. To evaluate cell diameter and capsule size, cells were stained with India ink and photographed on an Olympus AX70 microscope (Olympus America). Cell size was established based on measurement of cell diameter, and relative capsule size was established by measuring the capsule diameter to cell diameter ratio. To assess buoyancy, 3 ml of stationary-phase YPD liquid cultures for each strain were pipetted into cuvettes and allowed to settle passively for 6 h. Photographs were taken hourly for each cuvette, and the displacement from the top of the cuvette was measured to generate a graph of the displacement rate over time, as previously described (68). To measure cell surface hydrophobicity, wild-type and aquaporin mutant cultures were analyzed using the microbial adhesion to hydrocarbons (MATH) assay as previously described (69). In short, cells were grown overnight in YPD liquid media for 2 days at 30 °C and washed with DPBS. Cell cultures were suspended in 3 ml of DPBS with an OD600 ∼0.3, mixed with 400 μl hexadecane (Sigma-Aldrich), vortexed for 1 min, and rested for 2 min. After the settling period, the hydrophobic layer and the bottom layer (aqueous layer) were transferred to a 96-well plate, and the OD600 was measured in the SpectraMAX 340 Tunable Microplate Reader (Molecular Devices Ltd).

### Analysis of cell body and capsule size in capsule-inducing minimal media

To induce polysaccharide capsule growth, a 2 days YPD culture was washed with DPBS, and 10 μl of culture was transferred into 5 ml of minimal media (7.5 mM glucose, 10 mM MgSO4, 29.4 mM KH2PO4, 6.5 mM glycine, and 3 μM thiamine-HCl, pH 5.5) and incubated for 3 days at 30 °C, on the Cel gyro Rotator (Thermo Scientific). For long-term analysis, cells were incubated in 5 ml of minimal media and 100 μl of tested cultures were taken every 2 days to visualize cell and capsule size.

Alternative versions of minimal media were prepared at pH 4.5, 5.5, 6.5, and 7. To evaluate cell diameter and capsule size, cells were stained with India ink and photographed on an Olympus AX70 microscope. Cell and capsule size were measured using ImageJ and plotted using GraphPad Prism 10.1.1.

### Growth curve analysis in minimal media at varying pH

To assess the effect of AQP1 overexpression on pH-dependent growth, H99 and AQP1-overexpressing *C. neoformans* strains were cultured in minimal medium adjusted to different pH levels (pH 4.5, 5.5, 6.5, and 7.0). A 1 ml aliquot of medium was distributed to 24-well plates and inoculated with 5 × 10^5^ cells/ml. Cultures were incubated at 25 °C with constant shaking. Optical density at 600 nm (OD_600_) was measured every 30 min over time using a microplate reader (Spectramax M5) to monitor growth kinetics. Each condition was tested in 3 independent biological replicates, and results were expressed as the mean OD_600_ values over time. Growth data were visualized and analyzed using GraphPad Prism 10.1.1.

### Cellular and population-level total reactive oxygen species analysis

To determine the total ROS between H99, *aqp1*Δ, *aqp1*Δ::AQP1, and AQP1 overexpression strains, overnight YPD cultures were washed twice with DPBS, then cells were treated with 50 μM menadione to generate superoxide stress for 3 h in DPBS in a rotary tube mixer. Cell cultures were washed and stained for 30 min at 37 °C with either 10 μM dihydroethidium (DHE, ThermoFisher Scientific D23107) for cellular analysis of total ROS or 5 uM CellROX (Invitrogen, C10444) for population analysis of total ROS. After two washes, DHE-stained cells were mounted on an agarose patch and imaged on Olympus AX70 microscope.

### Titan cells formation and intracellular visualization of Aqp1-GFP and vacuoles

Cells from a 2-day YPD culture were washed with DPBS. The cells were inoculated in titan stimulation medium or on standard MM using a low inoculum. For the first method, 1 × 10^5^ cells/ml were inoculated in a 24-well plate with 1 ml of DPBS with 10% Fetal bovine serum (FBS) and incubated for 72 h at 37 °C and 5% CO_2_ (32, 70). For the low-inoculum titan cell induction method, 1 × 10^3^ cells/ml were inoculated into 5 ml of MM in 14 ml bottom-round tubes for 4 days with agitation. The cells were then recovered, washed with DPBS, and stained with India Ink. To visualize the intracellular localization of cryptococcal aquaporin1, a 2-day YPD culture of the GPF-tagged aquaporin strain was washed with DPBS. Then, 1 × 10^5^ cells/ml were inoculated in a 24-well plate with 10% Fetal bovine serum (FBS) and incubated for 72 h at 37 °C and 5% CO_2_ to stimulate titan cell morphology, or cells were incubated for 72 h at 30 °C in MM to present standard size yeast cells. After incubation, the cells were washed twice with DPBS and observed using an Olympus AX70 microscope. Cell size for titan-like cells was measured using ImageJ and plotted using GraphPad Prism 10.1.1.

For vacuolar and cell wall staining, cell cultures were labeled with FM4-64 (1 μg/ml) and UVitex 2B (5 μg/ml) for 30 min at 30 °C. After incubation, the cells were washed twice with DPBS and observed using an Olympus AX70 microscope. Cell size for titan-like cells was measured using ImageJ and plotted using GraphPad Prism 10.1.1.

### Inductively coupled plasma mass spectrometry

Selected cryptococcal cultures were incubated in 5 ml of minimal media in the rotating wheel for 3 days. Cell cultures were pelleted and washed twice with TE buffer (10 mM Tris, 1 mM EDTA, pH 8.0), then twice with Milli-Q H_2_O. Washed cell pellets were lyophilized to remove excess liquid and digested in 20% HNO_3_ overnight at 85 °C in metal-free 15 ml conical tubes. Digest was diluted to 5% HNO3 and analyzed at the Molecular Characterization and Analysis Complex at the University of Baltimore County for Fe, Mn, Zn, Cu, Ca, K, Na, P, and Mg analysis *via* PerkinElmer NexION 300D with ICP.

### Growth analysis in the presence of copper and zinc

To evaluate the impact of Cu or Zn on growth, *C. neoformans* strains (H99, *aqp1*Δ, *aqp1Δ*::*AQP1*, and *AQP1* overexpression) were cultured in minimal medium with varying concentration of CuSO_4_ (5–0.156 mM) (Sigma) or ZnSO_4_ (5–0.156 mM) (Sigma). Growth assays were performed in a 96 wells microtiter plate with a biological triplicate, with continuous agitation at 30 °C, and optical density at 600 nm (OD_600_) was measured using a microplate reader (Spectramax M5). Growth curve plots were visualized using GraphPad Prism 10.1.1.

### Assessment of growth under oxidative stress and antioxidant treatment

To analyze whether oxidative stress may have a stimulative impact on growth rate, *C. neoformans* strains (H99, *aqp1Δ*, *aqp1Δ::AQP1*, and *AQP1* overexpression) were cultured in YPD liquid medium supplemented with different concentrations of menadione (0, 25, 50, and 75 μM). Growth assays were performed in microtiter plates with continuous shaking at 30 °C, and optical density at 600 nm (OD_600_) was measured every 30 min using a microplate reader (Spectramax M5) to generate growth curves. Growth curve plots were visualized using GraphPad Prism 10.1.1.

Evaluation of antioxidant supplementation on the formation of titan cells was performed in titan cell-inducing conditions. In short, cryptococcal cells were incubated for 72 h in titan-inducing conditions (PBS with 10% heat-inactivated FBS, 37 °C, 5% CO_2_), with or without 3 mM ascorbic acid (AA), to assess whether antioxidants can limit aquaporin-associated increases in cell size. After incubation, cell body diameters were measured under a microscope (Olympus AX70) using ImageJ software. Only cells exceeding 6 μm in diameter were included in the analysis. Cell size distributions were visualized using GraphPad Prism 10.1.1.

### Cellular and population level total ROS and DNA content analysis

To determine the total ROS between H99, *aqp1Δ*, *aqp1Δ::AQP1*, and *AQP1* overexpression strains, overnight YPD cultures were washed 2x with PBS, and cells were treated with 50 μM menadione to generate superoxide stress for 3 h in PBS in a rotary tube mixer. Cells were washed and stained for 30 min at 37 °C with either 10 μM dihydroethidium (DHE, ThermoFIsher Scientific D23107) for cellular analysis of total ROS or the cell-permeable ROS probe, 20 μM H_2_DCFDA (Invitrogen), overnight at ambient temperature for population analysis of total ROS. After 2x washes in PBS, DHE-stained cells were mounted on an agarose patch and imaged on a Leica THUNDER widefield microscope; H2DCFDA-stained cells were imaged with an Attune Nxt Flow Cytometer to capture population shifts with at least 100,000 cells between strains compared to the no-stain control. For quantification of relative DNA content, cells were fixed with 4% paraformaldehyde overnight and stained with 10 μg/ml of propidium iodide on a rotary tube mixer. Cells were washed 2× with PBS for analysis on an Attune Nxt Flow Cytometer to capture population shifts with at least 500,000 cells. Population-level shifts were quantified with FlowJo v10, while single-cell images of total ROS were quantified with ImageJ v1. The results were visualized using GraphPad Prism 10.1.1.

### Western blot analysis of Sod1

Cultures were grown in minimal media and washed twice in PBS. Cells were lyophilized overnight and resuspended in lysis buffer (10 mM NaPi pH 7.8,0.1% Triton X-100,5 mM EDTA,5 mM EGTA, 50 mM NaCl, 10% Glycol) followed by the lysis by bead blasting 10× cycles with intervals of 30 s 3000 rpm and 30 s off at 4 °C. BCA assay was used to standardize protein load. A loading control (Abcam anti-GAPDH, ab9485, 1:1000 dilution) was used to assay for loading control between samples. For SDS-PAGE, 4 to 12% Bis-Tris gels were used, and sample preparation for 80 to 100 μg of protein with lithium dodecyl sulfate (LDS) and 100 μM DTT as the reducing agent. A semi-dry transfer on an iblot2 system onto premade stacks with PVDF membranes was done (Invitrogen IB24002). The blot was blocked for 1 h in 5% blotto and probed overnight with anti-serum against Sod1 (1:5000). Imaging was done with chemiluminescence using SuperSignal West Pico PLUS substrate (Thermo Scientific) and captured on an Odyssey Licor Scanner.

## Data availability

All data presented in the manuscript are contained within the manuscript.

## Supporting information

This article contains supporting information.

## Conflict of interest

The authors declare that they have no conflicts of interest with the contents of this article.
